# Adding Value to Fruit Processing Waste: Innovative Ways to Incorporate Fibers from Berry Pomace in Baked and Extruded Cereal-based Foods—A SUSFOOD Project

**DOI:** 10.3390/foods4040690

**Published:** 2015-11-24

**Authors:** Harald Rohm, Charles Brennan, Charlotta Turner, Edeltraud Günther, Grant Campbell, Isabel Hernando, Susanne Struck, Vassilis Kontogiorgos

**Affiliations:** 1 Chair of Food Engineering, Technische Universität Dresden, 01062 Dresden, Germany; E-Mail: susanne.struck@tu-dresden.de; 2 Department of Wine, Food and Molecular Biosciences, Lincoln University, 7647 Lincoln, New Zealand; E-Mail: charles.brennan@lincoln.ac.nz; 3 Department of Chemistry, Lund University, 221 00 Lund, Sweden; E-Mail: charlotta.turner@chem.lu.se; 4 Chair of Environmental Management and Accounting, Technische Universität Dresden, 01062 Dresden, Germany; E-Mail: edeltraud.guenther@tu-dresden.de; 5 School of Applied Sciences, University of Huddersfield, Huddersfield HD1 3DH, UK; E-Mails: g.campbell@hud.ac.uk (G.C.); v.kontogiorgos@hud.ac.uk (V.K.); 6 Research Group of Food Microstructure and Chemistry, Universitat Politécnica de Valencia, 46022 Valencia, Spain; E-Mail: mihernan@tal.upv.es

**Keywords:** pomace, berry processing, cereal foods, baking, extrusion

## Abstract

This article communicates the set-up of BERRYPOM, a European research project established in the second call of the SUStainable FOOD Production and Consumption (SUSFOOD) network. The project deals with the by-product from berry processing, which is frequently recycled as animal feed, composted or utilized for biogas production. With BERRYPOM it is proposed to analyze the value of berry pomace, to optimize the recovery of bioactive compounds from pomace material, and to incorporate processed berry pomace in cereal-based foods to take advantage of nutritional benefits that originate from its fiber and the content of bioactive substances. Additionally, extraction methods will be evaluated to obtain products rich in phytochemicals, and the influence of processing steps on the antioxidant capacity of pomace will be analyzed. The fiber extracts will then also be utilized in different cereal-based foods and extruded products. As project outcome we expect a substantial increase of knowledge concerning fiber and phytochemicals extraction from berry pomace, its suitability for enhancing nutritional and sensory properties of cereal-based foods, and its effects on the sustainability of the food chain.

## 1. Introduction

The European juice industry produced in 2012 10.4 million tons juice and nectar (www.aijn.org). The fraction of residues that remains after juice processing ranges from approximately 15% for grapes to 50% for citrus [1]. Because of the low content of nutrients and digestible energy of these residues, the utilization as animal feed is limited, while the high amount of phytochemicals, especially in the pomace of soft berry fruits, complicates composting because of their antimicrobial activity. On the contrary, these properties make pomace beneficial to human nutrition. Recycling methods that add value to fruit processing residues are of great interest, and it can be expected that the overall profit from fruit processing may be increased by an efficient and sustainable waste stream-management.

The risk of non-communicable diseases increases with insufficient fruit and vegetable consumption [2]. It was shown that health benefits attributable to berries are, for example, related to cardiovascular health [3], the reduction of inflammation [4], or the modulation of intestinal microbiota [5]. Consequently, the incorporation of bioactive compounds from fruit processing residues in foods helps to increase the supply of valuable nutrients. Clinical trials on potential prevention of chronic diseases revealed that the complex mixture of phytochemicals in foods has additive and synergistic effects so it cannot be replaced by individual compounds in dietary supplements [6]. Therefore, the combined incorporation of bioactive substances and fiber from berry materials in foods seems to be a promising approach. Because health information significantly influences the purchase intentions of consumers [7], elaboration of attractive products will contribute to European health policy, as the range of processed foods that can ensure dietary adequacy will be increased. Berries have a generally positive image, so that their inclusion in cereal-based products is likely to be viewed as appealing.

It is generally known that a fraction of 25%–50% of food is discarded at some point during production and consumption [8]. For reducing residue devaluation along the processing chain, it is necessary to increase the producer’s awareness towards products that, until now, are regarded as waste, and to build systematic strategies to find new markets for value-added intermediate ingredients processed from that waste. Together with awareness that can be created in consumers, the utilization of processed berry pomace may contribute to more sustainable food utilization.

The SUSFOOD research program targets food chain optimization with respect to sustainability and efficiency: it covers processing, packaging and retailing, food services and consumer activities, and promotes a multidisciplinary approach. In the context of the food processing industry, waste reduction also means that innovative ways should be approached to add value to promising materials currently not utilized in human nutrition. The project therefore is based on the following assumptions:

- Value can be added to the processing of soft berry fruits, e.g., blackcurrant and redcurrant, bilberry, chokeberry, or rowanberry (grapes are not topic of this project) when strategies and technologies are developed that make berry pomace usable as a food ingredient.
- Foods naturally rich or enriched with fiber and phytochemicals in the diet contribute to the health status of the consumer.
- As shown by Material Flow Cost Accounting and other methods, strategies for adding value to berry pomace have a sound economic and ecological basis.

## 2. The Proposed Research Agenda

### 2.1. General Targets

**Figure 1:**
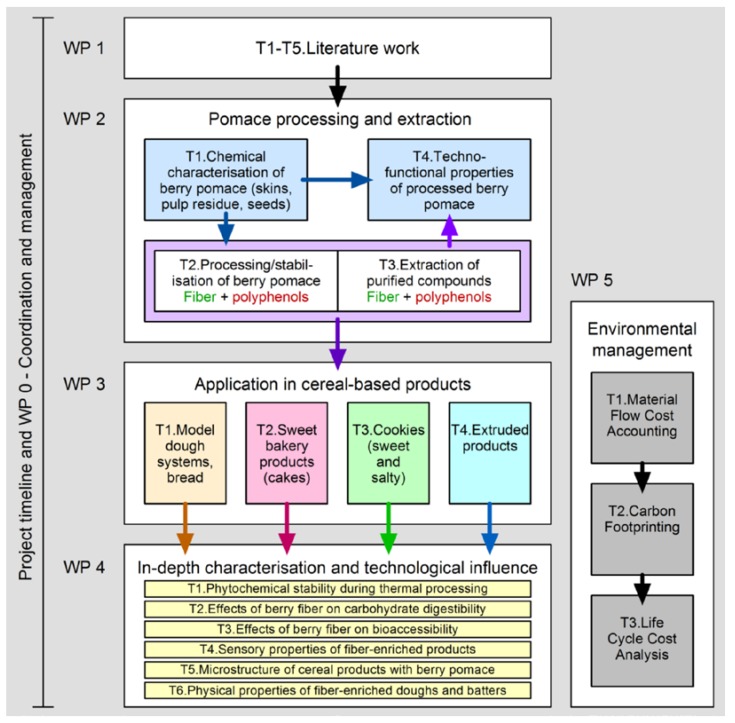
Research agenda of the BERRYPOM project. WP, work package; T, task.

In the project, we develop strategies to incorporate fiber and phytochemicals from berry pomace in cereal-based products. The two main approaches are (a) to determine the technical conditions that allow the delivery of an optimized product from fresh berry pomace with high polyphenols content and high shelf life, and (b) to modify and adapt formulations of cereal-based baked or extruded foods, which serve as targets for the incorporation of processed berry pomace. Material flow cost analysis gives information on the economic impact of this strategy (Figure 1).

Berries are soft sensitive fruits that can only be stored for a small period of time after a relatively short harvesting season. Apart from the 5–9 million tons of grape pomace per year that comes from worldwide wine production [1], it is also true for other berries that most of the harvest is processed in some form while only a minor amount is sold fresh or frozen. During juice processing of, for example, currant, raspberry or blueberry, approximately 20% remains as pomace. This material contains mainly berry skins, seeds and, sometimes, stems, and represents a valuable source of phytochemicals, pectin and dietary fiber. Through the incorporation of processed berry pomace in cereal-based foods:

- We take advantage of pressing residues from berry processing that would otherwise be discarded, and
- We increase the nutritional value of the target products because of the transfer of its fiber and beneficial phytochemicals.

In side tasks we will systematically evaluate whether processed berry pomace can be used to substitute sugar, flour or fat in baked products to obtain reduced energy foods.

### 2.2. Specific Approaches of the Research Proposal

#### 2.2.1. Pomace Processing and Extraction

The first part of the project delivers the basic compositional data for berry pomace of selected origin. Berries are rich in fiber from cell wall compounds, and in compounds with antioxidant capacity such as ascorbic acid, carotenoids and phenolics [9]. The respective aim is to develop procedures for the thermal and mechanical processing and fractionation of berry pomace to obtain functional fiber and other components of nutritional value. Methods for the production of non-perishable fiber extracts enriched with polyphenols will be elaborated, and processing parameters will be optimized. It is also necessary to analyze the influence of thermal and mechanical stress on antioxidant capacity and polyphenols content as, for example, the anthocyanins content in blueberry pomace is significantly reduced between 60 and 125 °C [10]. Rodriguez-Mateos *et al.* [11] also observed a heat-induced anthocyanins reduction in blueberries but no significant changes in the procyanidins content. Furthermore, processed berry pomace will be milled and size classified to determine the influence of the length of the fiber on its techno-functional properties.

Berry pomace is primarily composed of dietary fiber; in chokeberry and blackcurrant pomace, a content >90% in dry matter was observed [12]. The evaluation of the physical properties of the processed berry pomace (e.g., particle size, bulk volume, porosity, water and oil binding) will help to evaluate the technological value of the respective systems for their further use in cereal-based foods, and to establish the optimum conditions during processing.

#### 2.2.2. Application of Processed Berry Pomace in Cereal-based Products

In this section we incorporate processed pomace in different cereal-based foods (*i.e.*, bread, cakes, cookies, extruded snacks) to obtain fiber-enriched products. Cereal-based foods are an important fiber source, and the addition of supplemental pomace helps to achieve the recommended intake of dietary fiber. Fiber added to cereal-based baked or extruded foods also provides a technological bulking function [13] so that part of the sugar, flour or fat content may be reduced, and low-energy high-fiber bakery products can be provided for the consumer. A challenge is that problems may arise from changes in product characteristics: a decrease in volume and height, an increase in crumb hardness, both caused by altered microstructure and batter or dough rheology [14,15], or changes in appearance and taste [16,17]. Flavor and color of processed berry fiber have also to be considered. Consequently, the respective development of formulations has to be the first step to ensure a successful enrichment of cereal-based foods with this material. While clarifying the limits of processed pomace incorporation, the experiments will also deliver important information concerning the processing of the products before and during baking, and concerning their physico-chemical and sensory properties.

#### 2.2.3. In-depth Characterization of Processed Berry Pomace and Technological Influence

To gain information on the effects of berry pomace incorporation in cereal-based foods, fiber-enriched products will be analyzed. The nutritional relevance of berry pomace in these foods will be evaluated by focusing on starch digestibility and polyphenols bioaccessibility using *in vitro* digestion and enzymatic assays [18,19,20]. The amount of bioavailable phytochemicals with antioxidant capacity depends on the digestive stability, and the release from the food matrix [21]. A recent review concluded that the bioaccessibility of fiber-bound phytochemicals in the intestine is lower than the bioaccessibility of their extracted counterparts [22]. This will be verified by extraction and purification of polyphenols and fiber, and by individual addition to cereal-based foods. The effects will be compared to effects caused by the incorporation of processed berry pomace where polyphenols are bound to fiber. The extraction and analysis of polyphenols and other phytochemicals will be performed by “green” techniques using pressurized hot water [23], gas expanded liquids and supercritical CO_2_ as solvents [24,25]. Finally, we will be able to specify: thermal stability of phytochemicals during processing, the impact of processed berry pomace on carbohydrate digestibility and polyphenol bioaccessibility, and the implications on product microstructure. These data are essential for the transfer from scientific knowledge to the manufacture of cereal-based foods.

#### 2.2.4. Environmental Management

**Figure 2:**
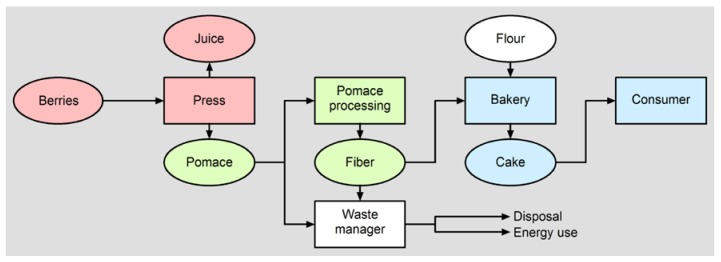
Flow chart for berry pomace incorporation in cereal-based foods.

We also interpret economic challenges and feasibilities that arise from strategies for processing berry pomace as (techno) functional ingredient in cereal-based products. The increase in berry processing sustainability is achieved by the reduction of processing waste, and process and resource efficiency by the re-use of organic by-products (Figure 2). Whereas recycling of pomace from berry processing helps to create additional benefit to up-to-now waste material and contributes to process efficiency improvement in the food manufacturing business, the production of fiber-enriched cereal products represents an innovative way to incorporate by-products in foods and to generate a product with additional nutritional value. Economic feasibility and environmental aspects of the re-use of pressing residues from berry processing will be assessed by performing Material Flow Cost Accounting, Carbon Footprinting, and Life Cycle Assessment [26,27].

## 3. Discussion and Expected Output

We expect developments that allow the tailored, value-added processing of berry pomace to be used in various types of cereal-based foods, improving their nutritional significance whilst reducing waste from berry processing. The utilization as food ingredient helps to improve the supply chain efficiency, and adds value to the berry processor, companies specialized in fiber or other functional food ingredients, the producers of cereal-based foods, and the health-conscious consumer. The project aims to establish new pathways for berry pomace by generating a model for the innovative transfer of a waste material into a food ingredient. This will increase resource efficiency of berry processing, and add to the consumer’s supply with healthy foods. The processing quality of the respective raw and intermediate materials, and chemical, physical and sensory quality of the final foods will be also considered. When necessary, technological drawbacks must be overcome by innovative formulation adjustment. The project therefore integrates research on the potential of a waste stream that is not regularly used as food, and on strategies to incorporate valuable materials in the human diet.

The transnational cooperation gives additional value to the project as implications on product characteristics can be evaluated in markets that differ in the food supply chain. To facilitate general conclusions, it is also possible to tailor formulations to local market requirements. The fact that we focus on pomace of different regional origin ensures that each contributing country is able to take its respective advantage.
